# The impact of polypharmacy and oral nutritional supplementation on nutritional status in patients residing in a long-term care facility

**DOI:** 10.3389/fnut.2025.1516103

**Published:** 2025-06-19

**Authors:** Lucyna Ścisło, Judyta Pluta, Anna Kliś-Kalinowska, Michał Górski, Marta Buczkowska

**Affiliations:** ^1^Institute of Nursing and Midwifery, Faculty of Health Sciences, Jagiellonian University Collegium Medicum, Kraków, Poland; ^2^Department of Occupational Medicine and Health in Department of Chronic Diseases and Civilization-Related Hazards, Faculty of Public Health in Bytom, Medical University of Silesia, Bytom, Poland

**Keywords:** nutritional status assessment, polypharmacy, oral nutritional supplements, older adults, nutritional status

## Abstract

**Introduction:**

Nutritional disorders and polypharmacy are among the most important problems facing the older adults in long-term care facilities. Polypharmacy negatively affects the nutritional status of the older adults. The use of specific drugs should be evaluated on a case-by-case basis, and a new drug should be introduced with caution. The supply of medications, especially among the older adults, needs more attention and analysis of their effects on nutritional status to avoid potentially dangerous consequences. The purpose of the study was to assess the nutritional status and determine the relationship between the pharmacotherapy used and the nutritional status of patients residing in a long-term care facility.

**Methods:**

The prospective, interventional study with a pre-post design, incorporating a retrospective study included 110 patients residing in a long-term care facility. Nutritional status was assessed before and after the nutritional intervention, which consisted of administering oral nutritional supplements (ONS). The study used the Mini Nutritional Assessment scale, body mass index (BMI), prealbumin, albumin, transferrin, and total lymphocyte count (TLC).

**Results:**

Studies conducted have shown malnutrition in a significant group of patients. The ONS used improved the assessed parameters of nutritional status. Multivariate regression analysis showed that the incidence of malnutrition is significantly influenced by variables such as gender, age, and polypharmacy. Polypharmacy negatively correlates with nutritional status.

**Discussion:**

There is an urgent need to improve nutrition practice and individual assessment of evidence-based pharmacotherapy use among older people, which is crucial to improving the quality of care provided.

## Introduction

Aging is a process in which the body gradually loses its natural ability to regenerate and function. In the process, a number of changes occur both at the cellular and whole-body level, which is associated with an increased risk of developing weaknesses and acute and chronic diseases (1).

One of the basic parameters for maintaining health is proper nutritional status. It depends on nutrient intake, utilization, and loss (2, 3).

The most common nutritional disorders include malnutrition, overweight, and obesity. These disorders are independent risk factors for new diseases, increase the risk of complications and mortality during treatment of other diseases regardless of the cause (4).

Their occurrence in the older adults is determined by factors such as loneliness, depression, social isolation, chronic diseases, pharmacotherapy, and unfavorable economic conditions (4, 5). The group of people most vulnerable to malnutrition are geriatric patients receiving long-term care (6, 7).

Nutritional deficiencies and low physical activity are common in the older adults due to poor health, disability, and low food intake. Sedentary lifestyle and negative net protein balance are also associated with aging influencing the occurrence of metabolic diseases, sarcopenia, and obesity, causing an increased risk of malnutrition and mortality in the older adults (8, 9).

It is estimated that nearly a quarter of people aged 65 and older are malnourished or at risk of malnutrition. As the world’s aging population increases, the problem is becoming more acute, and malnutrition of the older adults population is expected to increase (6, 7).

Maintaining proper nutritional status correlates with life expectancy and the effectiveness of treatment of underlying diseases. However, it is not always possible to maintain proper nutritional status through dietary interventions, so in some cases it is necessary to implement nutritional therapy through the use of oral nutritional supplements (ONS) (10, 11).

The European Society for Clinical Nutrition and Metabolism (ESPEN) defines ONS as products designed to provide energy and nutrients, including macro- and micronutrients (11, 12). In clinical practice, ONS are often used to support patients identified as malnourished or at risk of malnutrition (11, 13, 14). Some studies have shown that ready-to-use liquid ONS provide both clinical and economic benefits for a variety of patient populations in both hospitals and nursing homes (15, 16).

Early identification of older adults at high risk of malnutrition and provision of nutritional interventions is key to preventing malnutrition (17, 18).

A critically high risk of malnutrition is also found in some chronic conditions, dementia, and neurological diseases, especially in older people with multimorbidity (7, 19–21). Older adults living in institutionalized facilities with polypharmacy are particularly vulnerable (7, 21).

Polypharmacy, defined by the World Health Organization (WHO) as the use of five or more medications per day, is one of the significant problems affecting the geriatric population (22, 23). The older adults are taking more and more drugs, and analyses in various countries have shown that exposure to multi-drug use increases with age and the number of comorbidities (24, 25).

As a common and growing problem in older people receiving geriatric care, polypharmacy is associated with several complications, including those affecting nutritional status (24, 26, 27). The WHO introduced the term Medication-Related Harm (MRH), noting the growing public health problem of the sequelae of multiple medications, inappropriate prescribing, drug dosage, and treatment side effects, and drug-induced iatrogenic syndromes (22, 23, 28). It has been postulated that drug-dependent harm in the older adults should be considered a geriatric syndrome (22, 29, 30).

The polypharmacy phenomenon may also be the factor that reduces appetite leading to reduced food intake and deficiencies of essential nutrients (4, 6, 31, 32).

Polypharmacy is one of the potentially modifiable but unexplored and rarely observed factors affecting nutritional status, especially in older adults nursing home residents (33, 34).

There is not enough research providing strong evidence supporting the idea that polypharmacy affects the development of nutritional disorders. However, demographic projections show that there will be a significant increase in the number of people over the age of 65 in the coming years, which will lead to an increased need for pharmacotherapy to treat and prevent diseases in the older adults. There is a need to provide evidence on the impact of polypharmacy on nutritional status and its consequences.

## Objectives

The aim of the study was to assess the nutritional status before and after the introduction of nutritional intervention and to determine the relationship between the pharmacotherapy used and the nutritional status of patients residing in long-term care facility.

Before conducting the study, the following hypothesis was adopted:

**H:** Nutritional status negatively correlates with polypharmacy but positively correlates with supplementation ONS.

The hypothesis was verified by analyzing the collected data and performing statistical tests.

## Patients and methods

### Study group

The prospective, interventional study with a pre-post design, incorporating a retrospective study included 110 patients residing in a long-term care facility in Krakow.

Participation in the study was voluntary. Respondents gave independent and informed consent to participate in the study.

The study complied with the provisions of the Declaration of Helsinki and was approved by the Jagiellonian University Bioethics Committee No. KBET/59/B/2014.

The main criteria for inclusion in the study and inclusion of ONS supplementation were the patient’s existing risk of malnutrition as assessed by the Mini Nutritional Assessment (MNA) scale ranging from 17 to 23.5 points, no ONS supplementation to date, the patient’s status in a particular long-term care facility throughout the study period, consent to participate in the study and consent to the use of foodstuffs for special nutritional purposes at the time of the diagnosis of nutritional disorders, the absence of any previous nutritional interventions and the absence of symptoms of gastrointestinal disorders in the form of diarrhea or vomiting.

The study excluded people with cancer, disorders of consciousness, people under treatment for acute illness with present objective and subjective symptoms of infection, CRP > 10 mg/dl, people with acute positive symptoms associated with schizophrenia were also excluded from the study.

All patients included in the study group received verbal and written information about the subject, the purpose of the study, and the possible benefits of ONS supplementation.

### Research tool

The study used the method of analysis of medical records and interview (information was collected on age, gender, number of medications taken per day, and comorbidities).

To assess nutritional status used screening tests and laboratory results.

The data used in the study were collected according to the principles of full confidentiality and data storage rules. Data were recorded in the patient’s medical records, to which only medical staff and the patients themselves had access. The records were kept in a special archive, in locked cabinets.

Among the screening tests used to assess nutritional status were:

- Mini Nutritional Assessment – a scale that assesses the degree of malnutrition risk in the older adults, which is a combination of physical examination and nutritional history. The MNA questionnaire consists of a set of 18 questions with a maximum score of 30 points. The score was interpreted according to the following standard of points obtained (33): good nutritional status > 24; malnutrition risk 17–23.5; malnutrition < 17.
- Body mass index (BMI), according to the basic WHO classification, was interpreted according to the following guidelines (35): underweight < 18.5; normal value from 18.5 to 24.99; overweight ≥ 25.0.

The values of laboratory indicators of nutritional status were interpreted according to the following criteria (36):

- Transferrin (mg/dl): normal nutritional level > 200; mild malnutrition 150–199; moderate malnutrition 100–149; severe malnutrition < 100.
- Prealbumin (g/L): normal nutritional level > 0.15; mild malnutrition 0.1–0.15; moderate malnutrition 0.05–0.099; severe malnutrition < 0.05.
- Albumin (g/L): normal nutritional level > 35; mild malnutrition 31–35; moderate malnutrition 25–30.99; severe malnutrition < 25.

The total lymphocyte count (TLC) was calculated based on the formula (36):


TLC=%lymphocytes×leukocytecount/100.

Results were interpreted according to the following TLC/1 m^3^ standards: normal nutritional level > 1,500; mild malnutrition 1,200–1,500; moderate malnutrition 800–1,199; severe malnutrition < 800.

Blood samples were collected from patients according to laboratory procedures. Four ml of blood was drawn into tubes without anticoagulant for determination of prealbumin, albumin, and transferrin levels. Another 2 ml of blood was then drawn into tubes with EDTA containing edetic acid for hematological analysis to determine the level of total lymphocytes.

Nutritional status was assessed twice before and 2 months after nutritional intervention. The analysis included measurement of weight, height, and calculation of BMI, as well as MNA scale scores and laboratory parameters: transferrin, prealbumin, albumin, and TLC.

Nutritional status was assessed in 138 patients, and a select group of 110 patients at risk of malnutrition was finally included in the study, according to the MNA scale.

The study used standard ONS supplementation of 100 ml.

Oral nutritional supplements were administered as directed by a qualified nurse, who ensured that the patient took the full dose of the supplement. The adoption of the supplement was recorded in the patient’s individual record to monitor compliance and assess continuity of supplementation.

The energy value was 1,029 kJ/245 kcal; fat (35 En%) 9.6 g including: saturated acids 0.86 g;

Carbohydrates (41 En%) 25.1 g including: sugars 13.7 g; lactose < 0.35 g; fiber (0 En%) 0 g; protein (24 En%) 14.6 g; salt 0.09 g;

Vitamins: vitamin A 260 μg, vitamin D 2.08 μg, vitamin E 4.90 mg (α-TE), vitamin K 18.9 μg, thiamine 0.52 mg, riboflavin 0.56 mg, niacin (4.12 mg NE) 0.70 mg, pantothenic acid 1.53 mg, vitamin B6 0.61 mg, folic acid 80.9 μg, vitamin B12 0.90 μg, biotin 10.1 μg, vitamin C 30.7 mg;

Minerals and trace elements: sodium 35.0 mg, potassium 97.6 mg, chloride 60.0 mg, calcium 350 mg, phosphorus 182 mg, magnesium 32.5 mg, iron 3.85 mg, zinc 2.90 mg, copper 0.43 mg, manganese 0.84 mg, fluoride 0.18 mg, molybdenum 24.0 μg, selenium 14.7 μg, chromium 16.0 μg, iodine 36.3 μg;

Other: choline 88.1 mg, osmolarity 790 mOsmol/L.

The subjects took the formulations as prescribed twice a day for 14 days, following recommendations to drink at a slow pace (at least 2 h for one formulation).

A classification of the polypharmacy used among study participants was adopted (22):

- No polypharmacy – less than 5 medications or supplements taken simultaneously by the patient per day;
- Moderate polypharmacy – 5–10 medications or supplements taken concurrently by the patient per day;
- Heavy polypharmacy – 10 or more drugs or supplements taken concurrently by the patient per day.

### Statistical analysis

The normality of the distributions was assessed using the Shapiro–Wilk test. To assess the significance of differences, we used the χ^2^ test, Student’s t-test for dependent samples, and the Wilcoxon test. A nonparametric correlation test, including Cramer’s V correlation coefficient, was employed to analyze the relationship between the study variables. Results with *P* < 0.05 were considered statistically significant. To perform logistic regression analysis, the patients studied were divided into two distinct categories, using the presence of malnutrition as a cutoff criterion. A state of malnutrition was defined as a situation in which at least one of the selected blood biochemical parameters (transferrin, prealbumin, albumin, and lymphocyte count) was below an established reference level. Polypharmacy results were divided into two categories: presence of polypharmacy (including moderate and severe polypharmacy) and no polypharmacy. Univariate logistic regression analyses were performed to determine the factors relevant to the occurrence of malnutrition, which were used to build a multivariate model. Variables with *P* < 0.1 in the LR test were included in the model. Finally, variables with *P* < 0.05 on the Wald test (pWd) were considered to be factors significantly increasing the chance of a negative test result. The fit of the model was checked using the Hosmer-Lemeshow (pH-L) test (*P* > 0.05 no basis for rejecting H0 about a good fit of the model to the data) and based on the area under the graph of the ROC curve – the AUC (area under curve) value. Statistical analysis was conducted using Statistica 13.3 PL software (StatSoft Poland, Krakow, Poland).

## Results

The study group was predominantly female (70.1%). The smallest group consisted of those aged 61–70 (19.1%), and the largest were those aged 81–90 (32.7%). More than half of the respondents were characterized by moderate polypharmacy. Details of m distribution are shown in Table 1.

**TABLE 1:** Characteristics of the study group.

Variable	Total *N*; %	Sex		*P*-value^a^
		Women *N*; % 79; 70.1%	Men *N*; % 32; 29.9%	
**Age (years)**				
61–70	21; 19.1	8; 10.3	13; 40.6	0.15
71–80	29; 26.4	15; 19.1	14; 43.8	
81–90	36; 32.7	32; 41.1	4; 12.5	
91–100	24; 21.8	23; 29.5	1; 3.1	
**Polypharmacy**				
No polypharmacy (<5 medications/day)	43; 39.1	22; 28.2	21; 65.6	0.93
Moderate polypharmacy (5–10 medications/day)	62; 56.4	51; 65.4	11; 34.4	
Heavy polypharmacy (>10 medications/day)	5; 4.5	5; 6.4	0; 0.0	

*^a^*Chi-squared test.

Protein metabolism parameters: transferrin, prealbumin, albumin, and total lymphocyte count (TLC) were assessed before and after the introduction of ONS (Table 2). The mean values of each of the variables analyzed increased, but statistically significant increases were noted only for prealbumin and albumin.

**TABLE 2:** Evaluation of protein parameter values before and after oral nutritional supplementation.

Tested parameter	Classification	Total frequency before supplementation *N*; %	Mean (±SD) before supplementation	Total frequency after supplementation *N*; %	Mean (±SD) after supplementation	*P*-value^a^
Transferrin (mg/dl)	Severe malnutrition	5; 4.5	210.41 (±55.2)	6; 5.5	215.65 (±48.7)	0.07
	Moderate malnutrition	16; 14.5		7; 6.3		
	Mild malnutrition	33; 30		28; 25.5		
	Normal nutritional status	56; 51.0		69; 62.7		
Prealbumin (g/L)	Severe malnutrition	6; 5.5	0.16 (±0.60)	0; 0.0	0.18 (±0.58)	0.001
	Moderate malnutrition	17; 15.5		8; 7.3		
	Mild malnutrition	60; 54.5		61; 55.4		
	Normal nutritional status	27; 24.5		41; 37.3		
Albumin (g/L)	Severe malnutrition	6; 5.5	35.3 (±5.4)	4; 3.6	36.5 (±5.5)	<0.001
	Moderate malnutrition	21; 19.1		9; 8.2		
	Mild malnutrition	52; 47.2		31; 28.2		
	Normal nutritional status	31; 28.2		66; 60.0		
Total lymphocyte count (TLC) (number/mm^3^ of blood)	Severe malnutrition	3; 2.7	1468 (±137)	0; 0.0	1501 (±163)	0.39
	Moderate malnutrition	15; 13.6		10; 9.1		
	Mild malnutrition	16; 14.5		12; 10.9		
	Normal nutritional status	76; 69.2		88; 80.0		

***^a^***Wilcoxon test.

Every parameter analyzed shows improvement in protein nutrition in patients after implementing supplementation with special nutritional products.

Analysis of the screening scale for assessing nutritional status (MNA) shows an increase in the average score after reassessment, there was an increase of about 0.6 points. There was also an increase in the mean value of body mass index (BMI). Both parameters were statistically significantly different when comparing the study before and after supplementation (Table 3).

**TABLE 3:** Analysis of MNA and BMI values before and after oral nutritional supplementation.

Variables	Mean (SD)	*P*-value^a^
Mini nutritional assessment before the study	17.1 (±3.3)	0.001
Mini nutritional assessment after the study	17.7 (±3.6)	
Body mass index before the study (kg/m^2^)	19.8 (±3.2)	0.001
Body mass index after the study (kg/m^2^)	20.3 (±3.3)	

^a^Student’s *t*-test for dependent samples.

**FIGURE 1:**
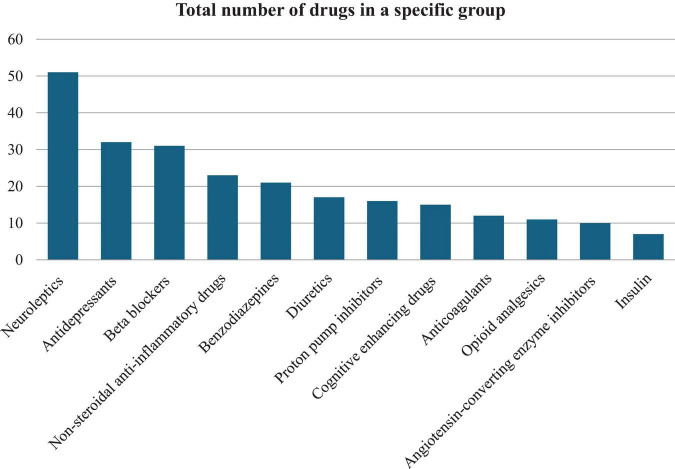
Analysis of the most commonly used medications among surveyed patients of a care facility.

**FIGURE 2:**
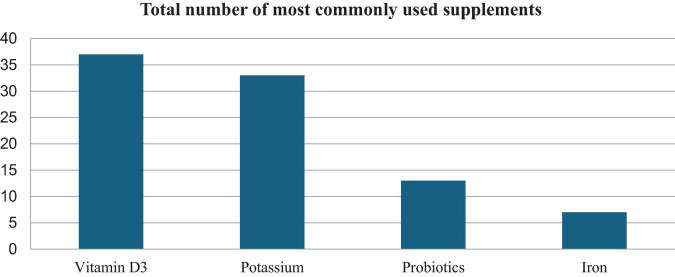
Analysis of the most commonly used dietary supplements.

Drug analysis identified the 12 most commonly used drugs. Neuroleptics were the most commonly used drug group, followed by antidepressants (Figure 1). Among dietary supplements, vitamin D3, and potassium were most commonly administered (Figure 2).

The statistical analysis carried out made it possible to isolate those groups of drugs that correlated statistically significantly with selected parameters of nutritional status. A negative correlation with TLC was shown for the use of neuroleptics (*P* = 0.048), proton pump inhibitors (PPIs) (*P* = 0.026), precognitive drugs (*P* = 0.01), and opioid analgesics (*P* < 0.001). On the contrary, the use of antidepressants (*P* = 0.032) and benzodiazepines (*P* = 0.042) showed a positive correlation with TLC. Benzodiazepines also positively correlated with albumin levels (*P* = 0.03). The use of procognitive medications, PPIs, and opioid analgesics negatively correlated with all assessed parameters of protein nutrition (Table 4).

**TABLE 4:** Relationship between medications used and selected nutritional status parameters before oral nutritional supplementation.

Group of drugs	Protein nutrition parameter	V-Cramer’s test	*P*-value^a^
Neuroleptics	Transferrin	−0.19	0.19
	Prealbumin	−0.16	0.84
	Albumin	−0.32	0.06
	TLC	−0.41	0.048
Antidepressants	Transferrin	0.42	0.08
	Prealbumin	0.15	0.12
	Albumin	0.47	0.07
	TLC	0.21	0.032
β-Blockers	Transferrin	0.16	0.25
	Prealbumin	0.23	0.09
	Albumin	0.19	0.62
	TLC	0.17	0.89
NSAIDS	Transferrin	0.10	0.64
	Prealbumin	0.32	0.36
	Albumin	0.21	0.086
	TLC	0.39	0.079
Benzodiazepines	Transferrin	0.23	0.51
	Prealbumin	0.18	0.26
	Albumin		0.03
	TLC	0.41	0.042
Diuretics	Transferrin	0.22	0.37
	Prealbumin	0.14	0.92
	Albumin	0.43	0.091
	TLC	0.27	0.62
Proton pump inhibitors	Transferrin	−0.35	0.047
	Prealbumin	−0.28	0.034
	Albumin	−0.37	<0.001
	TLC	−0.45	0.026
Procognitive drugs	Transferrin	−0.22	0.021
	Prealbumin	−0.31	0.036
	Albumin	−0.38	<0.001
	TLC	−0.24	0.01
Anticoagulants	Transferrin	0.27	0.52
	Prealbumin	0.36	0.31
	Albumin	0.21	0.17
	TLC	0.35	0.098
Opioid analgesics	Transferrin	−0.26	0.021
	Prealbumin	−0.23	0.0001
	Albumin	−0.34	<0.001
	TLC	−0.69	<0.001
Angiotensin convertase inhibitors	Transferrin	0.14	0.85
	Prealbumin	0.25	0.47
	Albumin	0.24	0.62
	TLC	0.29	0.32
Insulin	Transferrin	0.32	0.09
	Prealbumin	0.12	0.36
	Albumin	0.19	0.42
	TLC	0.35	0.32

^a^V-Cramer’s test.

Multivariate regression analysis showed that the incidence of malnutrition is significantly influenced by variables such as gender, age, and polypharmacy. The odds ratio (OR) for gender was about 1.7, meaning that women were about 1.7 times more likely to be malnourished compared to men. For the polypharmacy variable, the OR coefficient was almost 6, indicating an almost sixfold higher risk of malnutrition in patients with polypharmacy. In addition, the analysis showed that with each additional year of age, the risk of malnutrition increased by 13% (Table 5).

**TABLE 5:** Factors affecting the incidence of malnutrition (multivariate logistic regression).

Determinant		OR^a,b^ (95% CI)	pWd
Matching the model: p^H–L^ = 0.87; AUC = 0.779			
Sex	Male	Ref.	Ref.
	Female	1.69 (1.11–3.45)	0.013
Age (years)		1.13 (1.06–9.47)	0.0001
Polypharmacy	No	Ref.	Ref.
	Yes	5.88 (2.64–9.91)	0.001

AUC, area under the plot of the ROC curve; p^Wd^, Wald test; p^H–L^, Hosmer-Lemeshow test; CI, confidence interval; SE, standard error. *^a^*Reference group (OR = 1.0). *^b^*OR standardized to variables included in the model.

## Discussion

The nutritional status of older people depends on a number of factors, including physiological, social, economic, and psychological factors. Deteriorating health associated with age may contribute to inadequate food intake, therefore leading to malnutrition (31, 32, 35, 37).

Our research showed that a significant proportion of patients at the health facility were malnourished. According to transferrin levels, malnutrition was demonstrated in 49% of subjects, according to albumin levels in 71.8% of subjects, and according to prealbumin levels, 76.5% of subjects were malnourished.

The number of properly nourished individuals increased after the nutritional intervention among residents whose nutritional status was assessed by MNA, BMI, transferrin, albumin, prealbumin, and TLC. The mean level of the values of the individual nutritional status indicators after the nutritional intervention increased statistically significantly for MNA *P* = 0.001, BMI *P* = 0.001, prealbumin *P* = 0.001, and albumin *P* < 0.001. The authors’ studies on the use of ONS in the older adults suggest that clinical outcomes, such as mortality and complication rates, may be improved in older people with reduced nutritional status (15, 16). Studies by other authors have shown positive effects of ONS on weight gain (21, 38) and nutritional status as assessed by MNA and albumin levels, as well as physical fitness and quality of life (15, 16, 35, 37). These results confirm that implementing nutritional interventions can be a way to improve nutritional status and quality of life.

Maintaining good nutritional status is extremely important in order to reduce multi-morbidity and thus ensure the best possible quality of life. This article presents the impact of the use of specialized nutritional supplements on nutritional status and assessed the relationship between the pharmacotherapy used and the nutritional status of patients residing in a long-term care facility.

Research reports indicate that the phenomenon of multidrug use is a factor in unintentional weight loss among older people (21). Many drugs have been shown to cause side effects affecting the onset of malnutrition (20, 21). The studies also found an association between the use of certain medication groups and deteriorating nutritional status in older people (21, 38).

The large number of drugs used by patients is a contributing factor to malnutrition. Side effects are caused by some drugs, such as nausea and vomiting, diarrhea, dry mouth, or taste disturbances, resulting in reduced food intake. Available studies have shown that multidrug regimens containing more than 10 drugs cause malnutrition in the older adults within 3 years of use (39).

Studies highlight the association between polypharmacy and malnutrition, and the link between malnutrition and the occurrence of adverse drug reactions, in particular analgesics, benzodiazepines, PPIs, and cardiovascular drugs (21, 38). The same results were obtained in our own study. There was a statistically significant correlation between individual nutritional parameters assessed by albumin, prealbumin, transferrin, and TLC in relation to the drug groups used, such as PPIs. This means that, according to this group of drugs, there is a significant impact on the nutritional deterioration of older people. The available literature indicates that PPIs are of particular importance. Some studies suggest that long-term use of PPIs may reduce the absorption of vitamin B12, iron, magnesium, and calcium. Deficiencies of these substances can lead to various health problems, such as anemia or osteoporosis, which can affect nutritional status. Furthermore, the mechanism of action of PPIs is based on the inhibition of hydrochloric acid secretion by the cells lining the gastric mucosa. However, while a reduction in gastric juice acidity is beneficial in the treatment of gastrointestinal diseases, it can also affect the digestion and absorption of certain nutrients, especially proteins and minerals, which in the chronic aspect promotes the occurrence of malnutrition. Recent studies also suggest that long-term PPI use may lead to changes in the gut microbiome, which may also affect metabolic processes. In addition, it has been reported that a reduction in gastric juice acidity can reduce hunger or affect food taste and tolerance, as well as reduce appetite (12, 22, 39, 40).

Similar observations were reported for the use of opioid analgesics and procognitive drugs. Opioid drugs affect the central nervous system, including the arcuate nucleus and the ventral surface nucleus of the hypothalamus, which can lead to decreased appetite in some individuals. Furthermore, one of the most common side effects of opioid medication is constipation. Stopping intestinal peristalsis can lead to gastrointestinal discomfort, decreased appetite and reduced desire to eat. In addition to constipation, opioid drugs can also lead to other intestinal disorders such as nausea, vomiting, or diarrhea. These symptoms may affect the body’s ability to assimilate nutrients from food and predispose to nutritional status disorders (15, 22). Our own study also confirmed a statistically significant association between opioid medication use and nutritional deterioration in older people.

Particular attention should be paid to the use of procognitive drugs. Studies show that side effects of some substances in this group include reduced appetite, nausea, or changes in taste, which can make it difficult to maintain a healthy weight. The indicated symptoms may be considered as typical symptoms of dementia, but in some patients, they are due to the pharmacotherapy used (15, 38). In our study, procognitive drugs and neuroleptics negatively correlated with all assessed parameters of protein nutrition (albumin, prealbumin, transferrin, and TLC), meaning that they influenced the nutritional status of the subjects.

In our study, polypharmacy was common among the older adults population studied and as many as 12 different groups of medicines were used. The prevalence of malnutrition was significantly influenced by variables such as polypharmacy, but also by the gender and age of the subjects. Women were about 1.7 times more likely to be malnourished compared to men, and the risk of malnutrition was almost six times higher in polypharmacy patients. The analysis showed that the risk of malnutrition increased by 13% with each additional year of age.

Ramgoolie and Nichols (41) found that after adjusting for age and gender, older adults with polypharmacy assessed as six or more medications had significantly lower MNA scores than older adults without polypharmacy (OR: 3.94, 95% CI: 1.35–16.77, *P* = 0.015). The study considered factors that may influence incidence, such as age, gender, and number of diseases, and also included data on appetite complaints, changes in taste and smell, self-assessment of health, presence of nausea, and ability to swallow. Polyphagia and the number of complaints were positively associated with an increased risk of malnutrition. This result is statistically significant after taking into account age, gender, and number of diseases (*P* = 0.03) (41).

Farre et al. (42) study after adjusting for gender (female) and number of medications showed that there was a statistically significant association between (the risk of) malnutrition according to lower MNA score and more medications [(six or seven medications (OR: 3.23, 95% CI: 1.16–8.97, *P* = 0.02) and more than eight medications (OR: 5.58, 95% CI: 2.09–14.92, *P* = 0.001)] (42). A study by Maseda et al. (43) found that the use of more pre-scribed medications was associated with lower MNA-SF scores, and a logistic regression model for polypharmacy was significantly associated with (risk of) malnutrition (*P* < 0.001) (43). The study by Rodríguez-Sánchez et al. (44) found that the risk of malnutrition was significantly associated with increased medication intake (OR: 2.96, *P* < 0.05).

In our study, we demonstrated the association of polypharmacy on the occurrence of malnutrition. However, in light of the above findings of other authors, there is an emerging need for a study with a broad spectrum of multifactorial effects of polypharmacy on nutritional status.

The older adults also consume too few calories and essential nutrients. As a result, they face vitamin and mineral deficiencies, such as deficiencies of vitamin B2, vitamin B12, vitamin D, calcium, and iron (45). Our study included applied supplementation of vitamin D, potassium, probiotics, and iron. The problem of nutrient deficiencies occurring in the older adults raises the conclusion that all patients should receive nutritional counseling as a routine part of older adults care. Many symptoms and the very pathomechanism of diseases, especially in the older adults group increase the risk of malnutrition despite the need for pharmacological treatment of these conditions. Such a phenomenon in the face of taking many other medications, including often pharmacological supplementation not indicated or recommended prophylactically – can exacerbate malnutrition, especially in the older adults group. Therefore, monitoring the relationship between polypharmacy and nutritional status seems warranted to provide further evidence of the persistence of such abnormalities and to undertake preventive interventions.

It is important to raise awareness among caregivers of the older adults and medical personnel about the effects and negative consequences of polypharmacy and malnutrition. Prevention and timely assessment of polypharmacy, discharge, and monitoring of malnutrition can lead to a reduction in the risk of malnutrition in the older adults (45).

Our findings suggest that the appropriateness of specific drugs should be assessed on a case-by-case basis and a new drug should be introduced with caution. The supply of medicines, especially among the older adults, needs more attention and analysis of their impact on nutritional status to avoid potentially dangerous consequences. The team involved in the care of the geriatric patient must be adequately aware of the potential impact, both negative and positive, that drug therapy can have on nutritional status and overall health. Any assessment of nutritional status should include a review of the patient’s drug therapy and any opportunities to optimize it.

The study presented here focuses on assessing the nutritional status of patients in long-term care facilities and determining the relationship between medication use and nutritional status. Among the advantages of this study are the analysis of biochemical and immunological tests and the potential benefits associated with the use of food for special nutritional purposes. We consider that the assessment of the relationship between polypharmacy and nutritional status is an important issue that requires ongoing research, as it is still insufficiently investigated in the authors’ opinion. Nevertheless, we also note some negative aspects. This includes limiting the survey to a single city, making it impossible to relate the results to the whole of Poland.

We suggest that future studies be conducted in multiple centers, both nationally and internationally, to enhance the study’s quality and the reliability of its results.

The article also omits aspects related to potential cognitive and psychological confounders.

It is important to remember that mental health plays a crucial role and is strongly correlated with somatic health. Factors such as depression, social isolation, or cognitive decline could have influenced the overall results. Studies show (3, 6) that depressive episodes are often accompanied by a loss of appetite and aversion to food. Undoubtedly, changing life roles and reduced independence can increase the likelihood of depression in the studied patients, which may have impacted their nutritional status. Attention should also be given to the use of NMDA receptor antagonists (27, 28). Medications from this group can suppress appetite and alter eating behaviors, further affecting nutritional status. Additionally, polypharmacy can contribute to nutritional imbalances, as certain drugs may interfere with nutrient absorption, metabolism, or increase the risk of side effects that exacerbate malnutrition. PPIs are an example of such substances. Thus, careful consideration of both mental health and medication use is essential when assessing and managing the nutritional status of patients.

A limitation of the study is its short duration. We recommend that similar studies in the future last at least 6 months, which would enhance the reliability of the results.

There was also some limitation in conducting the study in long-term care facilities, which have limited diagnostic and therapeutic capacity in the Polish setting.

This includes limiting the survey to a single city, making it impossible to relate the results to the whole of Poland.

Despite the results of studies to date finding an association between polypharmacy and malnutrition, it is still unclear to what extent. There is a lack of detailed data on the number and type of medication intake associated with malnutrition. Based on such results, establishing clear criteria to limit the impact of polypharmacy on malnutrition in older people would help to reduce the potential risk of polypharmacy in clinical practice. On the other hand, for those at risk of malnutrition (due to the risk of polypharmacy), the introduction of nutritional support will be important evidence, resulting in the best possible quality of life.

There is a need for further research that takes into account the multifactorial effects of the drugs used, interactions between the drugs used, and excluding the effects of drugs that may lead to nutritional disorders. We plan to conduct a study with a control group in the future.

Monitoring the nutritional status of patients on polypharmacy is essential to identify potential nutrient-drug interactions and the risks of malnutrition that may arise from complex medication regimens. Basic anthropometric measurements (e.g., weight, height, BMI, and arm and calf circumference) should be assessed monthly, while laboratory markers (e.g., albumin, prealbumin, or TLC) should be evaluated every 3 months, or more frequently if necessary. A comprehensive approach, combining clinical evaluation with dietary intake analysis, is necessary for detecting deficiencies or imbalances in individuals taking multiple medications. Healthcare professionals should also consider the effects of specific drugs on nutrient absorption, metabolism, and utilization when monitoring nutritional status. Structured screening tools, such as the Malnutrition Universal Screening Tool (MUST), are frequently employed to assess the risk of malnutrition and guide appropriate nutritional interventions in polypharmacy populations. It is important to note that such care enhances patients’ quality of life and improves their overall health.

It is important to raise awareness among caregivers of the older adults and medical personnel about the effects and negative consequences of polypharmacy and malnutrition. Prevention and timely assessment of polypharmacy, discharge, and monitoring of malnutrition can lead to a reduction in the risk of malnutrition in the older adults (45).

## Conclusion

Studies conducted have shown malnutrition in a significant group of patients residing in a long-term care facility.

The ONS used improved the assessed parameters of nutritional status.

A significant number of patients show polypharmacy, which negatively correlates with nutritional status.

There is an urgent need to improve nutrition practice and individual assessment of evidence-based pharmacotherapy use among older people, which is crucial to improving the quality of care provided.

## Data Availability

The original contributions presented in this study are included in this article/supplementary material, further inquiries can be directed to the corresponding author.
